# Influence of Atomic Hydrogen, Band Bending, and Defects in the Top Few Nanometers of Hydrothermally Prepared Zinc Oxide Nanorods

**DOI:** 10.1186/s11671-016-1800-3

**Published:** 2017-01-06

**Authors:** Mubarak J. Al-Saadi, Salim H. Al-Harthi, Htet H. Kyaw, Myo T.Z. Myint, Tanujjal Bora, Karthik Laxman, Ashraf Al-Hinai, Joydeep Dutta

**Affiliations:** 1 Department of Physics, Sultan Qaboos University, PO Box 36, Al Khoudh, 123, Muscat, Oman; 2 Chair in Nanotechnology, Water Research Center, Sultan Qaboos University, PO Box 17 Al Khoudh, 123, Muscat, Oman; 3 Department of Chemistry, Sultan Qaboos University, PO Box 36, Al Khoudh, 123, Muscat, Oman; 4 Functional Materials Division, Materials and Nanophysics, ICT School, KTH Royal Institute of Technology, Isafjordsgatan 22, SE-164 40 Kista, Stockholm Sweden

**Keywords:** ZnO, Band bending, Surface defects, Hydrogen treatment, Visible light photocatalysis

## Abstract

We report on the surface, sub-surface (top few nanometers) and bulk properties of hydrothermally grown zinc oxide (ZnO) nanorods (NRs) prior to and after hydrogen treatment. Upon treating with atomic hydrogen (H*), upward and downward band bending is observed depending on the availability of molecular H_2_O within the structure of the NRs. In the absence of H_2_O, the H* treatment demonstrated a cleaning effect of the nanorods, leading to a 0.51 eV upward band bending. In addition, enhancement in the intensity of room temperature photoluminescence (PL) signals due to the creation of new surface defects could be observed. The defects enhanced the visible light activity of the ZnO NRs which were subsequently used to photocatalytically degrade aqueous phenol under simulated sunlight. On the contrary, in the presence of H_2_O, H* treatment created an electronic accumulation layer inducing downward band bending of 0.45 eV (~1/7th of the bulk ZnO band gap) along with the weakening of the defect signals as observed from room temperature photoluminescence spectra. The results suggest a plausible way of tailoring the band bending and defects of the ZnO NRs through control of H_2_O/H* species.

## Background

Zinc oxide (ZnO) is a wide band gap semiconductor material with a band gap of about 3.4 eV and a large exciton binding energy at room temperature (60 meV) [1, 2]. It has unique optical and electrical properties and can be grown in various morphologies using low-cost synthesis techniques [3]. It has been reported that well-ordered ZnO grains with fewer defects show better optical properties compared to the large discrete islands or structure less overgrowth based on flat continuous layers [4]. Microshape dependency shows that ZnO nanorods (NRs) have the best optical properties among nanoshells and nanoneedles [5]. In contrast, it has been also reported that enhanced surface defects play a crucial role in ZnO nanostructures when it is used as a visible light photocatalyst [6–9].

The method to enhance the optical properties of ZnO NRs has involved annealing in air [10], hydrogen treatment [11], and annealing in various environments [12, 13]. Yanob et al. showed that hydrogen is responsible for the near-band edge enhancement and drastic increment in the conductivity of the ZnO nanowires [11]. Ching-Ming Hsu et al. reported that the conductivity of thin films of molybdenum-doped zinc oxide was increased by a factor of 4 when treated by hydrogen over a period of 30 min [14]. Furthermore, hydrogen treatment of ZnO NRs can be used to control the electronic and optical properties of ZnO by creating defects within the ZnO crystal [15, 16]. These defects can be in the form of oxygen vacancies which act as deep donors, zinc vacancies as deep accepters, zinc interstitials as shallow donors, oxygen interstitials as deep accepters at the octahedral site, oxygen anti-sites as deep acceptors, and zinc anti-sites as shallow donors [17]. However, existence of a certain type of defect depends on the surrounding environment during the preparation or post-treatment of the ZnO NRs. Additionally, the formation and density of these defects is partly dependent on the chemical composition of the samples, wherein recent studies have shown that the presence of H_2_O can play a part in directing the defect formation [18]. A recent study by Gutmannet et al. [19] demonstrated the effect of annealing, ambient exposure, and photon flux-induced artifacts on work function *Φ* measurements of the nanocrystalline ZnO surfaces. The authors used ultraviolet photoemission spectroscopy (UPS) and low intensity X-ray photoemission spectroscopy (LIXPS) to determine the absolute *Φ* values and to confirm the hypothesis that surface hydroxylation by photo-induced H_2_O dissociation is most likely responsible for 0.30–0.35 eV *Φ* reduction of the band gap observed during UPS measurements. Their results suggest that any UPS measurements on ZnO surfaces exposed to ambient or H_2_O should consider 0.30–0.35 eV correction factor to determine the *Φ* accurately. Another study by Kumar Kumarappan [20] reported on the effect of H* cleaning on single ZnO (0001) crystal and associated upward band bending after partial removal of the surface contaminants at elevated temperature. Heinhold et al. [21] showed the influence of polarity and hydroxyl termination on the band bending at ZnO surfaces. Their results indicated how the Fermi level (*E*_f_) could be reversibly cycled between the conduction band and the band gap (*E*_g_) by controlling the surface H coverage using simple ultrahigh vacuum (UHV) heat treatments up to 750 °C, dosing with H_2_O/H_2_ and atmospheric exposure. In addition, they demonstrated the upward and downward surface band bending (*V*_sbb_) upon annealing of the H_2_O/H_2_ dosed O and Zn-polar faces of ZnO single-crystals.

Despite of the aforementioned efforts, hydrogenation of ZnO leads to effects which are not yet fully understood and explored. For example, in addition to the ambiguous electronic effects attributed to the hydrogen treatment of ZnO, it is unclear how the intrinsic H_2_O content due to the ZnO NRs soft chemistry preparation interacts with H* in the top few nanometers at room temperature—this is different from the abovementioned studies which were based on annealing of the H_2_O/H_2_ dosed O and Zn-polar faces of ZnO single-crystals. In addition to that and to the best of our knowledge, there are no reports on the effects of the electronic structure variation—such as band bending—in pristine- and hydrogen-treated ZnO NRs.

In this work, we report on the preparation of ZnO NRs on glass substrate by a simple hydrothermal process. The effect of trapped H_2_O molecules on the band bending at the surface of the ZnO NRs is probed using XPS and UPS. The effect of H* treatment on the samples with and without intrinsic H_2_O molecules is also explored in terms of band bending and defect-induced photoluminescence (PL) intensities. The correlation between oxygen vacancies with PL intensities and valence band maximum (*E*_VBM_) of ZnO NRs with and without trapped H_2_O molecules are further discussed.

### Experimental

#### Materials

The ZnO NRs were synthesized using the following materials: Analytical grade zinc acetate dihydrate (Zn (CH_3_COO)_2_·2H_2_O) was purchased from MERCK, Germany, and zinc nitrate hexahydrate (Zn (NO_3_)_2_·6H_2_O) and hexamethylenetetramine ((CH_2_)_6_N_4_) were obtained from Sigma-Aldrich, USA. All the chemicals were used without further purification. Standard microscope glass slides were used as substrates for the growth of ZnO NRs, which were cleaned in ultrasonic water bath using soap water, acetone, ethanol, and deionized (DI) water prior to the growth of the nanorods.

## Methods

### Substrate Seeding with ZnO Nanocrystallites

ZnO nanocrystallites were seeded on glass substrates using 10 mM solution of zinc acetate dihydrate in 20 mL dissolved in DI water followed by spraying on pre-heated (350 °C) substrates [22, 23]. After spraying, the samples were annealed at 350 °C for 5 h in the ambient and stored in an oven at 90 °C until further use. The purpose of ZnO seeding was to augment nucleation sites for ZnO NRs growth [24, 25].

### Hydrothermal Synthesis of ZnO NRs

ZnO NRs were grown on the pre-seeded glass substrates by following facile hydrothermal process as reported in previous works [26, 27].

Briefly, the seeded glass substrates were placed in a solution with equimolar concentrations (20 mM) of zinc nitrate hexahydrate and hexamethylenetetramine (precursor solution) and then kept in an oven at 90 °C. The growth process was carried out for 10 h, and precursor solution was replenished after 5 h to maintain a constant growth rate for the nanorods [26]. Then, the samples were thoroughly rinsed with DI water and annealed at 350 °C in ambient air to remove any un-reacted chemicals on the surface. The positioning of the glass slide inside the furnace and annealing temperature were crucial to engineer portions of ZnO NRs with and without H_2_O on the same glass slide simultaneously. These portions were identified by XPS before commencing of any experiment.

### Atomic Hydrogen Treatment

**Fig. 1:**
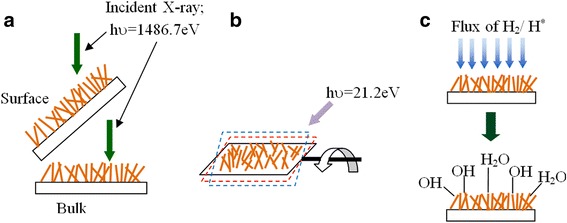
**a** XPS surface and bulk geometry. **b** UPS bulk geometry is shown, and surface geometry is obtained by tilting the sample. **c** Atomic hydrogen cracking process

Hydrogen treatment was carried out using hydrogen cracking cell from Omicron (Fig. 1c). The efficiency of cracking was almost 10%. The hydrogen gas pressure was kept stable at 10^−6^ mbar for 2 h which resulted in the sample hydrogen exposure of about 7.2 KL (1KL = 7.2 × 10^−3^mbar.s).

### Characterization

Surface morphology of ZnO NRs on glass substrates was characterized by JEOL JSM-7800F (Japan) field emission scanning electron microscope (FESEM) working at 30 kV. X-ray photoemission spectroscopy (XPS) (Omicron Nanotechnology, Germany) with a monochromatic Al Kα radiation (hν = 1486.6 eV) working at 15 kV was used for surface, sub-surface, and bulk analysis of ZnO NR samples before and after the hydrogen treatment. Figure 1a shows experimental geometry used for XPS investigations. The obtained XPS spectra were deconvoluted to individual components using Gaussian Lorentzian function with Casa XPS software and calibrated with respect to the C 1s feature at 284.6 eV. During the XPS experiments, all the measured samples were flooded with electrons to neutralize surface charging effects. Ultraviolet photoelectron spectroscopy (UPS) (Omicron Nanotechnology, Germany) with radiation energy of (21.2 eV) was used for surface and sub-surface density of state analysis of ZnO nanorods samples before and after hydrogen treatment by changing the sample tilt angle as depicted in Fig. 1b. In order to measure changes in the ZnO *Φ* due to hydrogen treatment, UPS was calibrated and tested using ITO thin film following the procedure adopted by Park et al. [28]. X-ray diffraction (XRD) of ZnO NRs were obtained by using a Rigaku MiniFlex600 X-ray diffractometer with Cu Kα X-ray radiation (wavelength = 1.54 Å). Room temperature photoluminescence (PL) of the NRs were recorded in a Perkin Elmer LS55 fluorescence spectrometer with an excitation wavelength of 325 nm. Photocatalytic activity test was carried out on H* untreated and H* ZnO samples soaked in 10 ppm phenol solution. The phenol degradation was done under simulated solar light (AM 1.5 radiation, 1 kW/m^2^), and the phenol degradation kinetics were studied by using ultra performance liquid chromatography (UPLC, LC-30AD, Shimadzu, Tokyo, Japan) technique.

## Results and Discussion

**Fig. 2:**
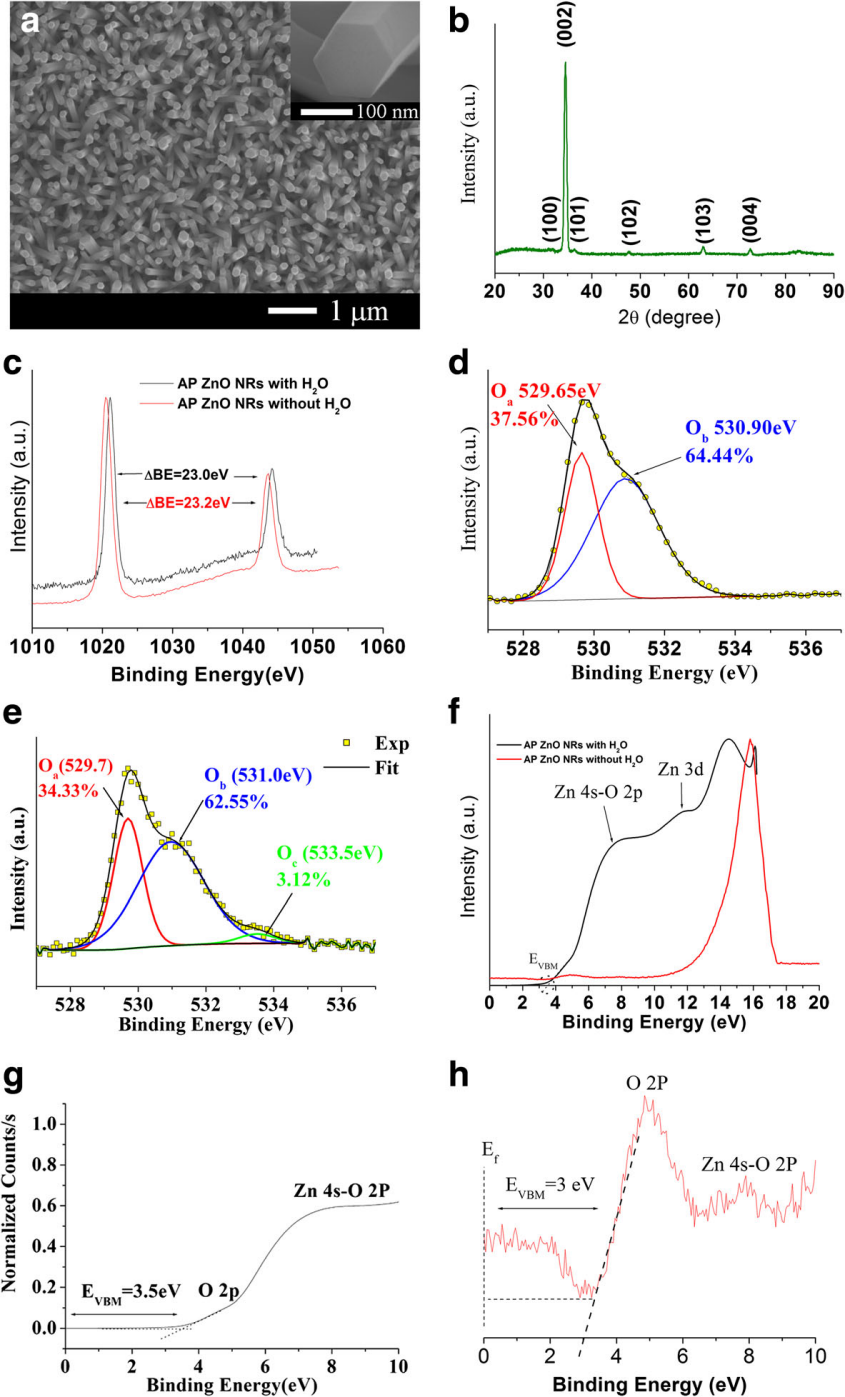
**a** Scanning electron micrograph of ZnO NRs (*inset*: magnified image of the hexagonal structure of a single crystal ZnO nanorod). **b** XRD spectrum of ZnO NRs. **c** Zn 2p XPS spectrum of the as prepared (AP) ZnO NRs with and without H_2_O. **d** O 1s XPS spectrum of ZnO without H_2_O. **e** O 1s XPS spectrum of ZnO with H_2_O. **f** UPS spectra of ZnO NRs with and without H_2_O samples. **g** UPS valence band region of ZnO with H_2_O. **h** UPS valence band region of ZnO without H_2_O

Scanning electron micrographs of as-prepared ZnO NRs grown on glass substrates were observed to have an average diameter and length of 120 ± 10 nm and 3.5 ± 0.2 μm, respectively (Fig. 2a). X-ray diffraction (XRD) analysis of the as-grown samples showed the hexagonal wurtzite structure of the ZnO crystal as confirmed by the 2*θ* values at 31.8, 34.4, 36.2, 47.4, 62.9, and 72.7 (JCPDS card no. 01-089-0510) corresponding to (100), (002), (101), (102), (103), and (104) crystal planes, respectively, as observed in Fig. 2b [26, 29]. The strongest XRD peak at 34.35° indicates the preferred growth of the rods along (002) crystal direction. Some of the as-prepared ZnO NRs were observed to have chemisorbed H_2_O and some did not as is indicated by an increase of 0.2 eV in XPS binding energy for the Zn 2p peak—for the sample with H_2_O—which results from the spin orbital splitting of the Zn 2p core ionization peak as shown in Fig. 2c [30].

To better understand the surface composition of the NRs, the oxygen (O 1s) peaks of the as prepared samples with and without chemisorbed H_2_O molecules were studied using XPS. As shown in Fig. 2d, asymmetric O 1s peak of the sample without H_2_O on the surface could be coherently fitted by two Gaussian components, centered at 529.7 eV (*O*_a_) and at 531.0 eV (*O*_b_). An additional component at 533.5 eV (*O*_c_) is found for the sample with H_2_O as shown in Fig. 2e, attributed to chemisorbed H_2_O species on NR surface. The *O*_a_ peak is attributed to lattice oxygen in wurtzite ZnO forming Zn–O bonding, while *O*_b_ is attributed to O^2−^ ions in oxygen-deficient regions within the ZnO matrix (oxygen vacancies) and the surface adsorbed loosely bonded oxygen like hydroxyls (OH) bonds, i.e., ZnO(OH) [31]. *O*_c_ can be ascribed to the specific chemisorbed oxygen, from adsorbed CO_2_, O_2_, or H_2_O [32]. The peak positions correspond well with literature, which show that the *O*_b_ and *O*_c_ peaks lie at approximately 1.35 and 3.8 eV to the right of the lattice oxygen peak in ZnO crystal [33]. In order to observe the effect of the chemisorbed H_2_O species on the electronic properties of ZnO NRs, ultraviolet photoelectron spectroscopy (UPS) technique was employed as shown in Fig. 2f. An enhancement in the intensity of band structure features (i.e., the valence band maximum (*E*_VBM_), Zn 4s-O 2p (~6–8 eV), and Zn 3d (~10.5–11.5 eV)) is observed for the ZnO sample which contains the chemisorbed species. Consequently, the *E*_VBM_ was found to be high (3.5 ± 0.1 eV) as estimated from the linear extrapolations of the foremost edges of the UPS data graphs depicted from Fig. 2g. It is noteworthy that the O 2p (~4.5 eV) ZnO-related surface state is absent in the UPS of the sample with H_2_O but visible for the samples without H_2_O with reduced *E*_VBM_ value of (3.0 ± 0.1 eV) as shown in Fig. 2h.

**Fig. 3:**
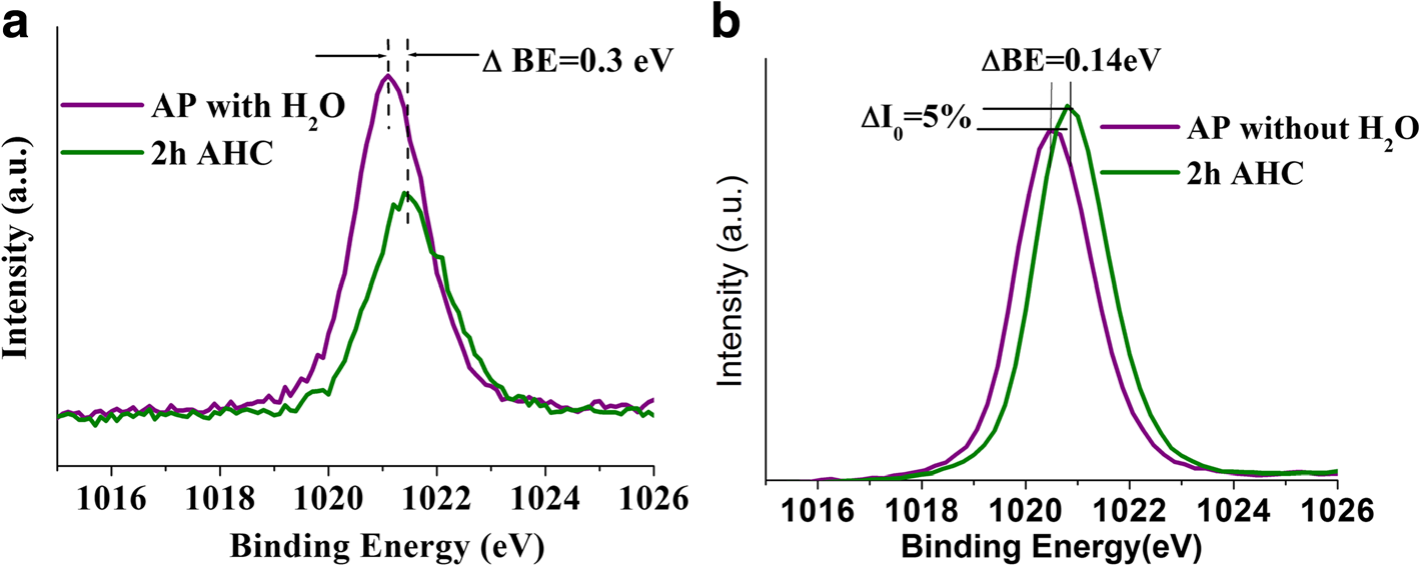
Normalized Zn 2p XPS spectrum of as-prepared ZnO NRs with (**a**) and without (**b**) H_2_O before and after hydrogen treatment for 2 h

ZnO defects were investigated on samples with and without adsorbed H_2_O species subjected to H* treatment for 2 h. Figure 3a, b shows theZn 2p core level ionization peaks after the H* exposure. It is clear from Fig. 3a that a significant reduction in the Zn peak is detected after hydrogen treatment for samples with H_2_O on the surface. The results suggest that H* either etches ZnO surface removing zinc ions from the crystal lattice or forms a layer on the surface of the rods which causes a reduction in the Zn intensity due to the reduction of the XPS sampling depth. The latter explanation is most likely responsible for the reduction of Zn peak intensity. This is supported by the Zn peak binding energy shift of 0.3 eV caused by the layer creating a surface potential (i.e., band bending) which is anticipated to change the *Φ* of the sample in the presence of surface H_2_O. On the contrary, hydrogen treatment of the sample without H_2_O shows both slight increase in the intensity (Δ*I*_o_ = 5%) of Zn and binding energy shift (0.14 eV) (Fig. 3b) suggesting cleaning process of ZnO surface by H* to occur.

**Fig. 4:**
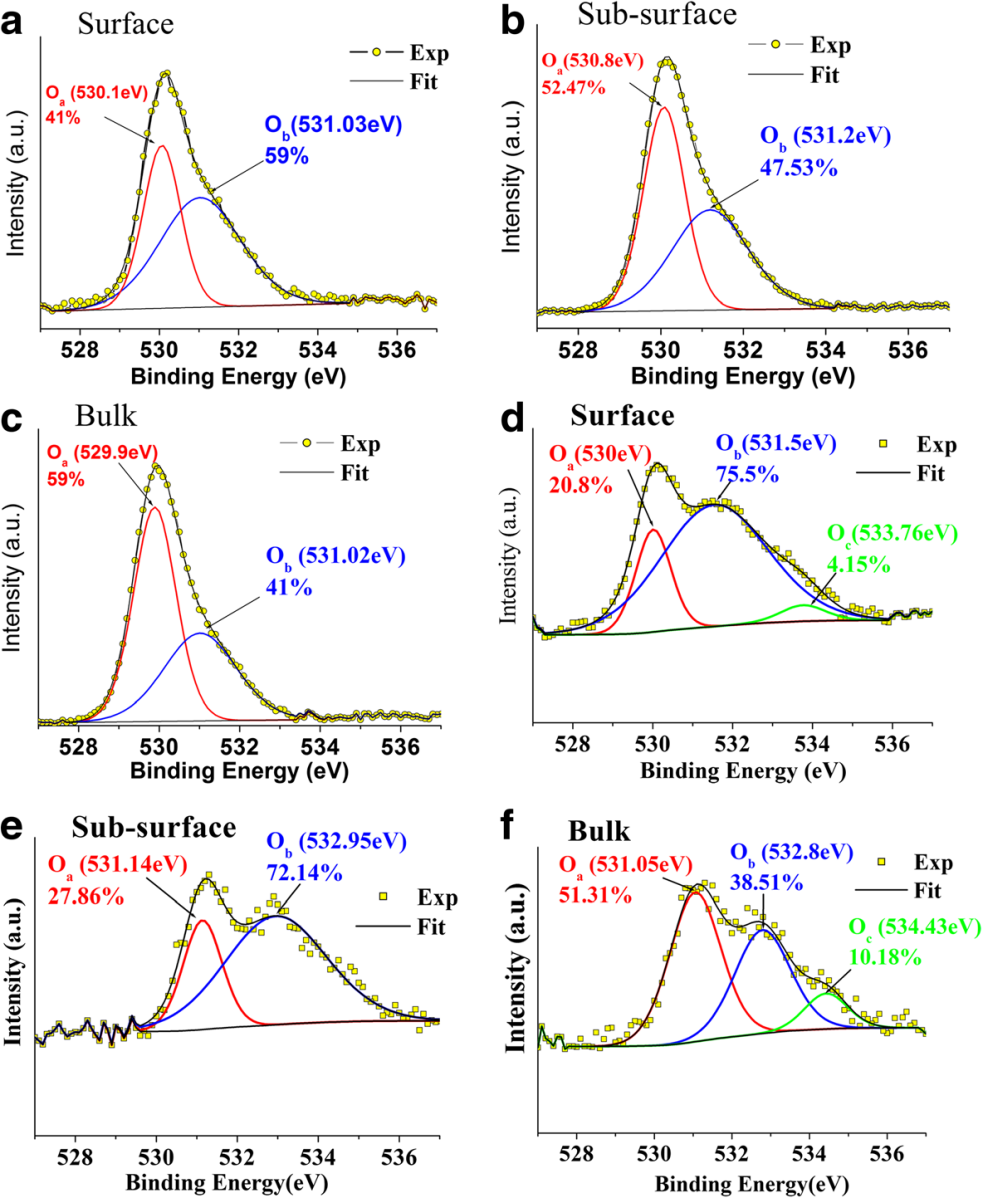
O 1s XPS spectra of ZnO nanorod samples treated with hydrogen in the absence of adsorbed H_2_O **a** surface, **b** sub-surface, and **c** bulk and with H_2_O **d** surface, **e** sub-surface, and **f** bulk

XPS depth profiling was carried out to get better insight of the distribution of defects in ZnO NRs treated by H*. The schematic diagram showing experimental geometry indicates incident X-ray direction on ZnO sample as shown in Fig. 1a. The bulk characterization of O 1s peaks was achieved with X-rays perpendicular to the ZnO surface, while glancing angles provided surface and sub-surface information. Figure 4a–c shows the O 1s peak of ZnO nanorods without H_2_O after treatment with H* for 2 h. Upon moving from the surface to deep-bulk, we observe that *O*_b_ decreases while *O*_a_ increases and not surprisingly, no adsorbed H_2_O species (i.e., *O*_c_ peak) was observed. Correspondingly, the O 1s signals obtained from the sample with H_2_O (Fig. 4d–f) show existence of *O*_a_, *O*_b_, and *O*_c_ components on the surface and in bulk regions. *O*_c_ depletion layer is also found at sub-surface region of the sample as seen in Fig. 2e. This confirms the presence of H_2_O (i.e., *O*_c_ ~ 4.15%) on the ZnO NRs surface and in the bulk ZnO crystal (*O*_c_ ~ 10.18%), sandwiching the depletion layer.

Auxiliary experiments (not shown here) of Ar ion beam sputtering of 5 keV or annealing at 500 °C for 1 h of the ZnO NRs lead to the removal effect of the bulk H_2_O content. A careful comparison between O 1s peaks for the sample without H_2_O after hydrogen treatment and before (i.e., comparison between Figs. 4a and 2d) reveals that H* treatment has an effect in the reduction of *O*_b_ (from 64.4 to 59%) and increase of the binding energy of the O 1s (from 529.7 to 530.8 eV for *O*_a_ and from 530.9 to 531.0 eV for *O*_b_), therefore supporting the cleaning effect to take place due to the H* treatment.

Despite an increase in the binding energy of O 1s obtained for the sample which contains H_2_O, *O*_b_ tends to increase as seen from the comparison between Figs. 2e and 4d. The increase in binding energy observed for O 1s (0.3–0.4 eV) and Zn 2p_3/2_ (0.2 eV) peaks after hydrogen treatment suggests weakening of the Zn–O bond in the crystal lattice, which can increase the nuclear attraction force experienced by the electron, resulting in the increase of binding energy for both lattice oxygen and zinc.

**Fig. 5:**
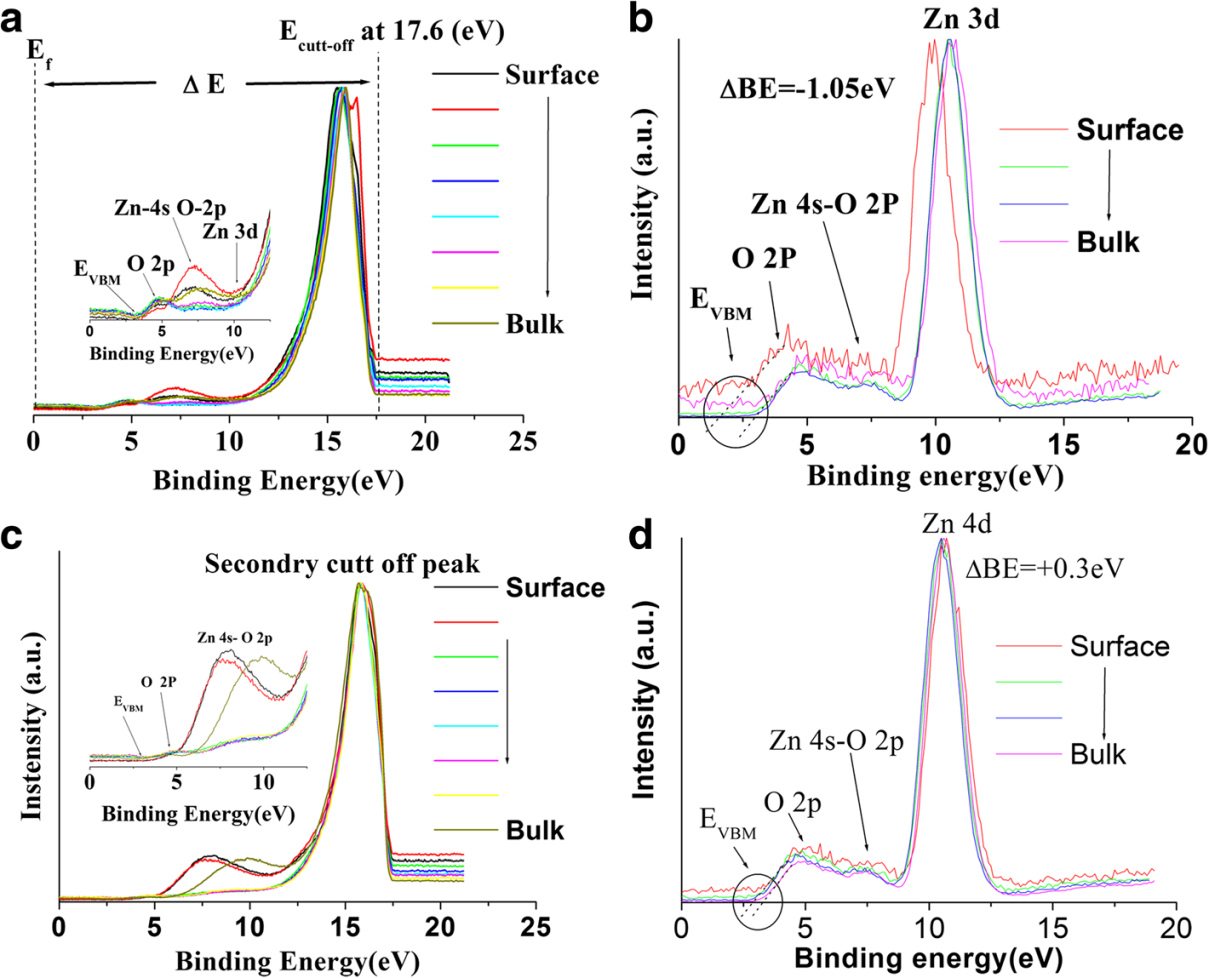
UPS and XPS valence band spectra from surface to bulk of **a** UPS of as-prepared ZnO NRs in the absence of H_2_O before atomic hydrogen cracking (AHC), **b** XPS of as-prepared ZnO NRs without H_2_O before AHC, **c** UPS of as-prepared ZnO NRs without H_2_O after AHC, and **d** XPS of as-prepared ZnO NRs without H_2_O after AHC. All UPS and XPS valence spectra after AHC are seen be less noisy and smooth compared to that of not being treated by hydrogen

The effect of hydrogen treatment on the electronic properties of samples without H_2_O and with H_2_O was investigated by UPS and by XPS valence data. Figure 5 shows the UPS and XPS valence band spectrum obtained for the sample without H_2_O. Before hydrogen treatment, all ZnO surface states (O 2p (~4.5 eV), Zn 4s-O 2p (~6–8 eV), and Zn 3d (~10.5–11.5 eV)) are detected as revealed in Fig. 5a. The inset of Fig. 5a shows slight variation from 3.3 to 2.8 eV for the *E*_VBM_ and attenuation of the Zn 3d peak. Taking the *E*_VBM_ value of 2.8 and 3.37 eV energy gap (*E*_g_) for ZnO [22], the surface band bending (*V*_sbb_) was calculated from

1 $$ V\mathrm{s}\mathrm{b}\mathrm{b} = E\mathrm{g}-{E}_{\mathrm{VBM}}-\varSigma $$

where *Σ* = (kT/*q*)ln(*N*_C_/*n*) [34] is the energy difference between *E*_F_ and the conduction band minimum (*E*_CBM_) in the bulk of the sample (*n* is the bulk carrier concentration 2 × 10^17^ cm^−3^ and *N*_C_ is the conduction band effective density of states = 2.94 × 10^18^ cm^−3^ for ZnO). Using these values, the *Σ* was found to be 0.064 eV and *V*_sbb_ = 0.51 eV. This positive *V*_sbb_ value is a sign of upward band bending which generates an electron depletion layer on the ZnO NRs surface and is comparable to the 0.53 eV value found by Kumarappan [20] upon H* cleaning of ZnO (0001) single crystal at high annealing temperatures. The UPS band structure features and the decrease in *E*_VBM_ values are supported by the XPS valence band spectra presented in Fig. 5b. Due to the glazing angle used for XPS investigation, it is observed that the Zn 3d XPS core level—not to be confused with the secondary cutoff peak of UPS shown in Fig. 5a and *E*_VBM_ shift to lower binding energy value of 1.6 eV as evident in Fig. 5b. This value is attributed to surface contaminants covering the un-treated surface. As depicted in the inset of Fig. 5c and upon hydrogen treatment, the UPS O 2p peak gets attenuated and the Zn 4s-O 2p appears with enhanced intensity as the main feature related to the hydrogen treatment of the ZnO sample without H_2_O. The trend of band structure features after hydrogen treatment is clearly supported by XPS valence band data shown in Fig. 5d. For example, *E*_VBM_ values estimated from Fig. 5d show a decrease from 3.3 to 2.8 eV moving from bulk to surface geometry. The large *E*_VBM_ value of 3.3 eV confirms an upward band bending and is close to the band gap (3.37 eV) of ZnO. Furthermore, this value is expected from the maximum XPS sampling depth (*d*) of ~10 nm estimated from 3*λ*_max_ = *d*, where *λ*_max_ is the maximum electron mean free path value equaling to 3.5 nm for the XPS Al Kα radiation used in this study. The decrease of *O*_b_, attenuation of the UPS O 2p peak, intensity enhancement of Zn 4s-O 2p, and enhanced smoothness of all UPS and XPS spectra after hydrogen treatment strongly strengthen the conclusion that hydrogen treatment does have a cleaning effect on the ZnO NR sample in the absence of H_2_O.

**Fig. 6:**
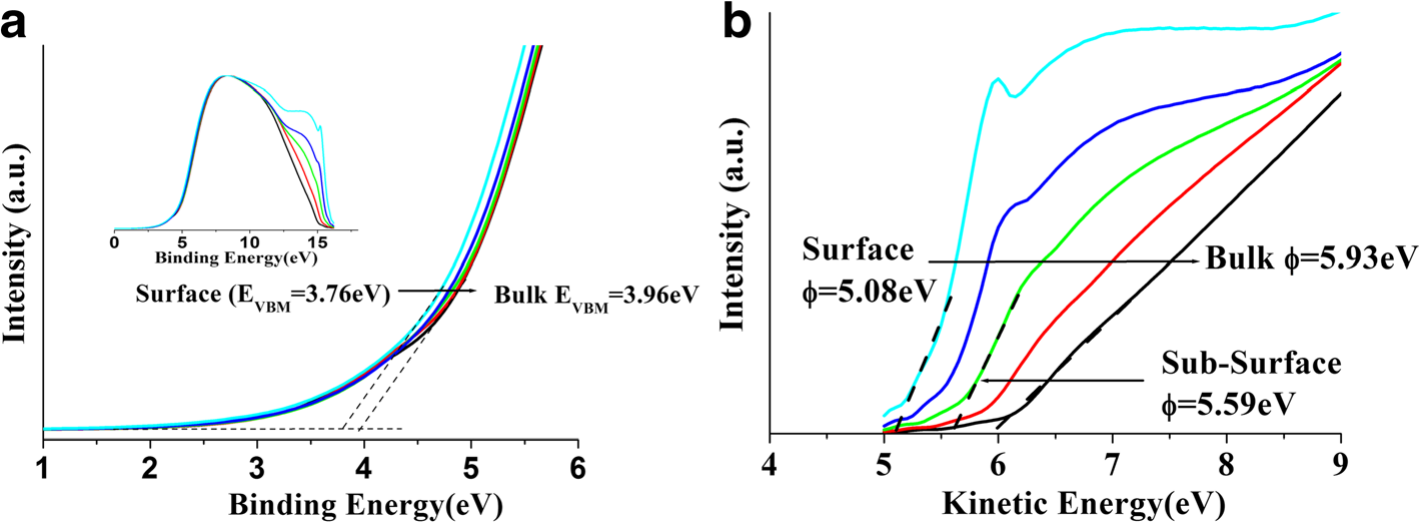
UPS spectra from surface to bulk of **a** *E*_VBM_ variation as estimated from UPS spectra. *Inset* shows the UPS spectra for the ZnO NRs with H_2_O after AHC. **b** Estimated *Φ* values from UPS spectra from surface to bulk of after AHC for ZnO NRs with H_2_O

Electronic band structure results obtained from the analysis of the UPS data after hydrogen treatment from the sample with H_2_O show different behavior from that without H_2_O. The sample with H_2_O shows that there are some energy states like Zn 4s-O 2p and Zn 3d (see black spectrum in Fig. 2f) which totally disappears after treatment with hydrogen for 2 h as shown in the inset of Fig. 6a. This could be because of the layer formed on the surface of the rods by hydrogen interacting with *O*_b_ mediated by the presence of *O*_c_ (i.e., adsorbed H_2_O). This interaction is manifested by an increase of 13% in *O*_b_ after hydrogen treatment as seen from the comparison of *O*_b_ content in Figs. 2e and 6a. *E*_VBM_ decreases from 3.96 to 3.76 eV from bulk to surface as shown in the inset of Fig. 6a. Employing Eq. (1) and using the *E*_VBM_ value of 3.76 eV and Eg = 3.37 eV, the *V*_sbb_ native value of 0.45 is observed—a sign of downward band bending and the development of electron accumulation layer at the sample surface.

The work-function (*Φ*) can be calculated from the difference in the photon energy of He (*I*) (21.2 eV) and the energy difference Δ*E* between the secondary cutoff energy (*E*_cutoff_) and the Fermi edge (*E*_F_) as shown in Fig. 5a as

2 $$ \varPhi =21.2-\varDelta E $$

The *Φ* values calculated using Δ*E* obtained from Fig. 5a, c are 3.6 ± 0.1 and 3.7 ± 0.1 eV before and after hydrogen exposure for the sample without adsorbed H_2_O.

To illustrate the effect of H* treatment on the work function (*Φ*) for the sample with adsorbed H_2_O, the UPS spectra in Fig. 6b are plotted with respect to the kinetic energy. Therefore, the extrapolated lines fitted on the secondary cutoff peaks to the energy scale directly correspond to the *Φ* values. Clearly, large *Φ* values (compared to that found in the sample without water) decreasing from 5.9 ± 0.1 to 5.1 ± 0.1 eV are found from the bulk to the surface, respectively. Considering the aforementioned correction factor (0.3–0.35 eV) following the work function of Gutmann et al. [19] attributed to the effect of UV exposure during the UPS experiments, the corrected surface *Φ* values after H* treatment turn to be 4.0 ± 0.1 and 5.4 ± 0.1 eV for the sample without H_2_O and with H_2_O, respectively.

**Fig. 7:**
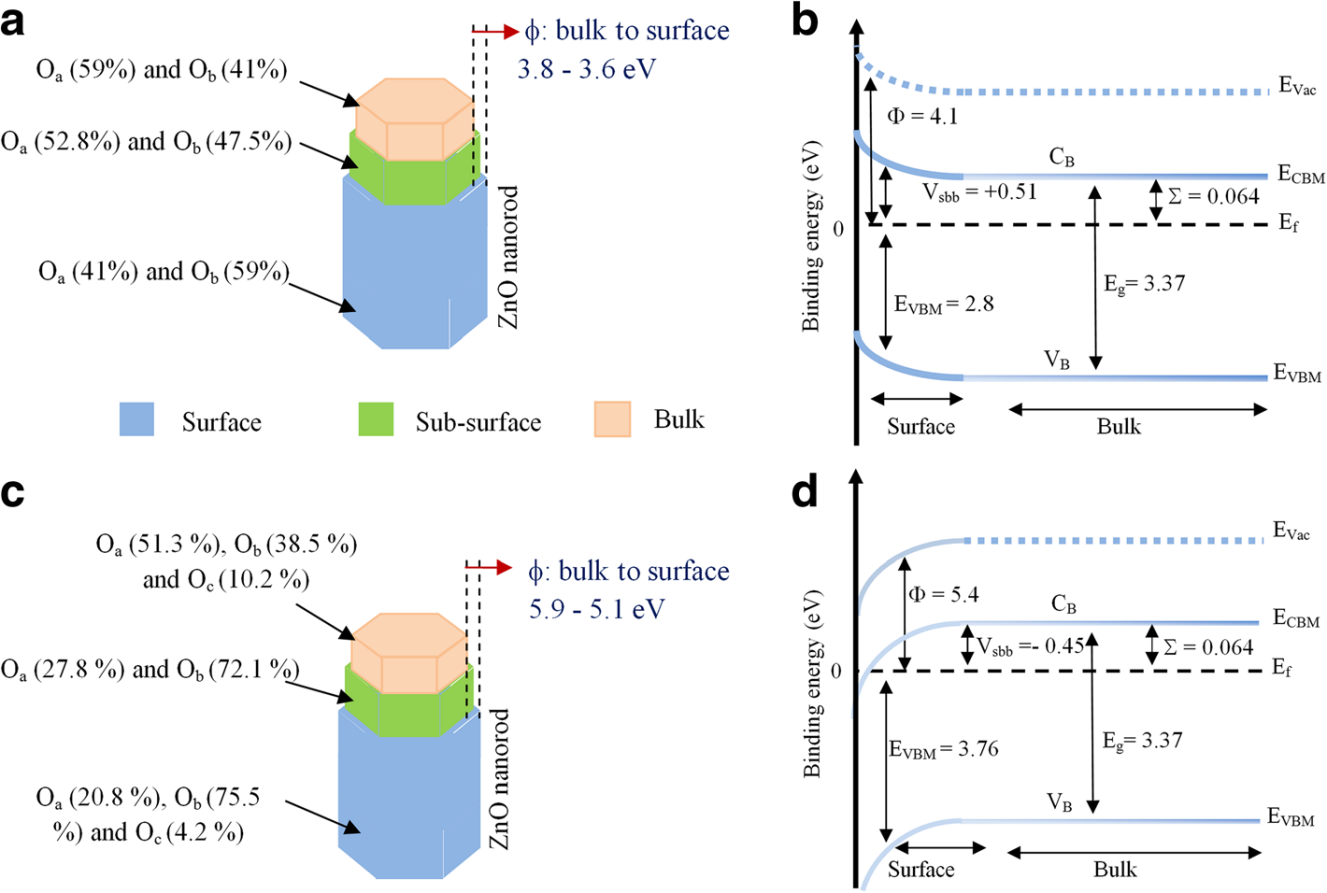
Proposed model of the *O*_a_, *O*_b_, and *O*_c_ distribution, *Φ* and band bending diagrams of ZnO NRs after treated by hydrogen for 2 h, **a** ZnO NR chemical composition without H_2_O, **b** band bending ZnO NRs without H_2_O, **c** ZnO NR chemical composition with H_2_O, and **d** band bending ZnO NRs with H_2_O. Note that *Φ* value variations from bulk to surface shown in **a** and **c** are not subjected to Gutmann et al. [19] 0.3–0.35 eV correction factor

Figure 7 shows the proposed model of the surface, sub-surface and bulk chemical composition, *Φ* values, and band bending diagrams for the ZnO NR samples with and without H_2_O content after hydrogen treatment.

It is interesting to compare the measured *E*_VBM_ and *Φ* values with those reported before. The *E*_VBM_ (2.8–3.3 eV) and *Φ* (4.1 eV) values found for the sample without water after H* treatment agree very well with published values by Kim et al. [35] (*Φ* = 4.08 eV) after Ar^+^ ion sputtering/heating ZnO single crystal at 700 °C, Gutmann et al. [19] (*E*_VBM_ = 3.0 eV, *Φ* = 4.1 eV) on nanocrystalline ZnO surfaces after annealing at 400 °C in UHV environment, and Heinhold et al. [21] (*E*_VBM_ = 3.41 eV) after annealing ZnO single crystal at 750 °C for 15 min. This agreement is not surprising since annealing or Ar^+^ ion sputtering has similar effect of partial cleaning of ZnO as H* treatment.

**Fig. 8:**
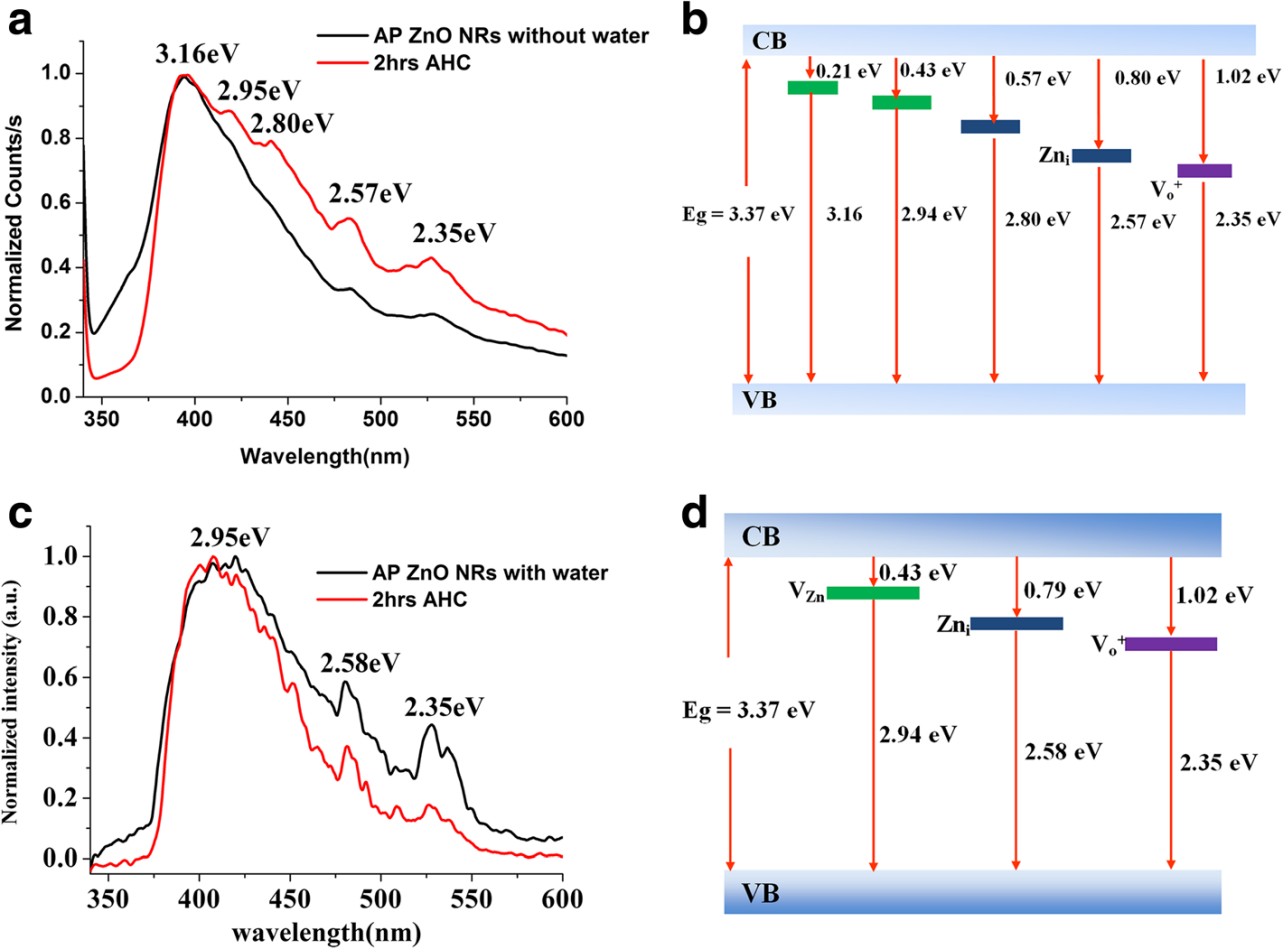
Room temperature photoluminescence spectra of the ZnO NRs before and after H* treatment. **a** PL for sample without H_2_O. **b** Energy position of defects in sample without H_2_O. **c** PL for sample with H_2_O. **d** Energy position of defects in the sample with H_2_O. The relative of the green emission intensity peak at 2.35 eV with respect to the UV emission is denoted as *R* value

Room temperature photoluminescence (PL) was recorded with the excitation wavelength of 325 nm for all samples before and after H* treatment. Figure 8a presents the data measured for the ZnO sample without H_2_O before and after H* treatment. It is evident that after hydrogen treatment, the intensity of all emission attenuated peaks is increased due to the removal of surface contaminates with the cleaning of ZnO NR surface. The strong emission peak around 421 nm (2.94 eV) can be assigned to the recombination of an electron at zinc interstitial (Zn_i_) and a hole in the valence band [36]. Two other peaks observed at 480 nm (2.58 eV) and 527 nm (2.35 eV) can be assigned as different defect state emissions [36]. Vanheusden et al. [37] had reported that the visible luminescence of ZnO mainly originate from different states such as oxygen vacancies Vo^0^, Vo^+^, and Vo^+2^ and Zn_i_. The oxygen vacancies are located below the bottom of the conduction band (CB) in the sequence of Vo^0^, Vo^+^, and Vo^+2^, from top to bottom. The peak around 527 nm can be related to singly ionized oxygen vacancy. While the green emission is a result of the recombination of the photogenerated hole with a singly ionized charged state of the specific defect. According to Anderson et al. [17] and Vanheusden et al. [37], emissions related to defects in our sample can be assigned to zinc interstitial at 480 nm (2.58 eV) as a shallow donor and singly ionized oxygen vacancy and at 527 nm (2.35 eV) as a deep donor. Zinc interstitial (Zn_i_) produces a shallow donor level at 0.79 eV below the bottom of CB, and the singly ionized oxygen vacancy produces a deep donor at 1.02 eV below the bottom of CB (see Fig. 8) [38–40]. A new strong UV emission peak is found around 390 nm (3.16 eV) attributed to ZnO–OH—supported by XPS data at binding energy of 531.2 eV in Fig. 4b—species on sub-surface of the ZnO sample without H_2_O. Upon hydrogen treatment of the H_2_O adsorbed on sample, the intensity of emission peaks in the visible range from 400 to 600 nm is reduced compared to what is observed for the un-treated sample. This reduction can be understood through a reaction of H + O^2−^ → OH^−^ + e^−^. The excess electrons from this reaction neutralize the positively charged oxygen vacancies thus reducing the visible PL intensity. Interestingly, the new UV peak observed at 390 nm (3.16 eV) in the sample without H_2_O after H* treatment is absent in the sample with adsorbed H_2_O molecules. The positions of different defect levels are schematically shown in Fig. 8b, d for the samples without H_2_O and with H_2_O, respectively.

It is very imperative to understand the relationship between band structure and the measured core level binding energies parameters and with the observed PL features. Table 1 shows a summary of PL *R* parameter and *E*_VBM,_ *V*_sbb_, and Zn core-level binding energy (BE) values obtained from UPS and XPS spectra, respectively. The *R* value is defined as the relative of the green emission—it can be any emission in PL spectra—intensity peak at 2.35 eV with respect to the UV emission.

**Table 1:** PL *R* factor, *E*_VBM_ and *V*_sbb_ UPS parameters, and XPS Zn binding energies

Sample type	No H* treatment	With H* treatment
Sample without H_2_O	*R* = 4.1, *E*_VBM_ = 3.0 eV	*R* = 2.3, *E*_VBM_ = 2.8 eV, *V*_sbb_ = 0.51 (upward)
	Zn (BE) = 1020.5 eV	Zn (BE) = 1020.8 eV
Sample with H_2_O	*R* = 2.2, *E*_VBM_ = 3.5 eV	*R* = 5.7, *E*_VBM_ = 3.76 eV, *V*_sbb_ = 0.45 (downward)
	Zn (BE) = 1021.1 eV	Zn (BE) = 1021.5 eV

The surface band bending phenomenon seen from *V*_sbb_ is correlated to the estimated PL *R* values. The upward band bending reflects small *R* value (2.3) (i.e., enhancement of PL intensity) for the sample without H_2_O. However, the downward band bending induced the negative accumulation layer in the sample with H_2_O, causing attenuation of PL (i.e., large *R* value 5.7).

**Fig. 9:**
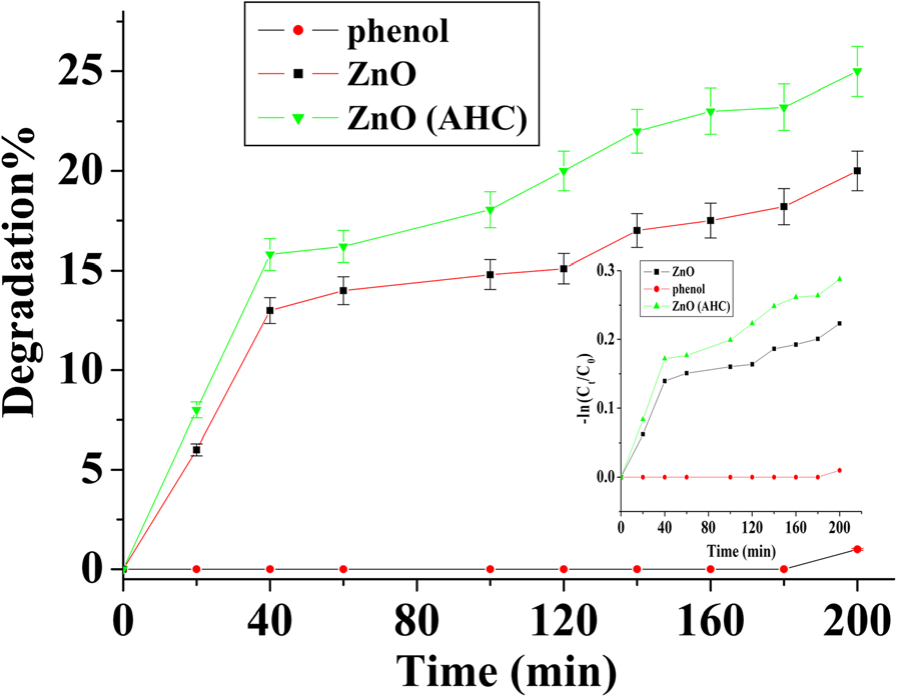
Visible light photocatalytic degradation kinetics of phenol with and without ZnO (H* treated and pristine) having different surface defects densities (*inset* shows the pseudo first kinetic degradation model for ZnO, ZnO (AHC), and without ZnO)

Based on PL results, the ZnO sample without H_2_O showed enhancement of defects after H* treatment. Therefore, this sample was used to study the photocatalytic degradation of phenol under solar light irradiation. The concentration of phenol at various time intervals as shown in Fig. 9 was calculated from the area under the phenol peak. In addition, the rate constant (*k*) was estimated from Fig. 9: Inset using first order pseudo kinetic model {−ln(*C*_t_/*C*_0_) = kt} where *C*_t_ is concentration of phenol at time *t* and *C*_0_ is an initial phenol concentration. It was observed that the degradation of phenol took place in two stages: The first stage from 0–40 min and the second stage from 40–200 min with *k* values of 0.0035 ± 0.0002 and 0.00050 ± 0.00005 for ZnO and 0.00430 ± 0.00008 and 0.00080 ± 0.00005 for H*-treated ZnO, respectively. After 180 min, 24% of phenol degradation was observed for the H*-treated ZnO compared to 18% degradation in the presence of pristine ZnO nanorod sample. As a result, H* treatment of ZnO at room temperature demonstrated 25% improvement in photocatalytic degradation of phenol attributed to the surface defects. It is anticipated that the photocatalytic degradation of phenol will be further enhanced for ZnO samples treated with H* at high annealing temperatures [41].

## Conclusions

Zinc oxide nanorods were synthesized on glass substrates using a hydrothermal process, and the surface defects were modulated by H* treatment at room temperature. XPS and UPS revealed the surface, sub-surface, and bulk chemical composition and electronic band structures of the ZnO samples with and without H_2_O in their structure. The H* treatment had the effect of cleaning the ZnO NRs, enhancement of PL signals, upward band bending, and improved phenol degradation for the sample without H_2_O. Downward band bending and attenuation of PL signal were the main features for the sample with H_2_O. The reported results show that the surface, sub-surface, and bulk chemical oxygen vacancies can be correlated to the observed defects and the H_2_O/H* species can be used to tailor the band bending of the ZnO NRs which might be required for several applications.
